# 

**Published:** 1838

**Authors:** 


### CONTENTS OF NO. XLV.

### ESSAYS AND CASES.

*Page.*

ART. I.—CLINICAL REPORTS:
Introductory remarks. 1
Obliteration of the Vagina cured by the knife, by Dr. Robson. 2
Facial Neuralgia cured with Belladonna, by the same. 3
Osteo Sarcoma of the lower jaw, by Dr. Wort. 5
Compound and Comminuted fracture running into the elbow joint, by Dr. Meek. 8
Peritoneal Enteritis by Dr. Barbee. 9
Permanent cure of Hernia with Chase's apparatus by Dr. Orr. 13
Encephalic disorder with Aphonia by Dr. Malone. 16
ART. II.—Medical Topography, climate and disease of Western New York by Dr. Hamilton. 17
ART. III.—Strictures and Inversions of the Uterus by Dr. White, in a letter from Dr. Kreider. 30
ART. IV.—Physiological researches by M. Gerdy; translated from the French by Dr. Warder. 35
ART. V.—Compensation of Physicians especially in the Western States by Dr. Drake. 49

### REVIEWS AND BIBLIOGRAPHICAL NOTICES.

ART. VI.—Velpeau's Clinical Lectnres on White Swellings, reported by M. Jeanselme; translated by Dr. Warder. 57
ART. VII.—Homes' Statistical and Pathological Reports on Phthisis Pulmonalis. 70
ART. VIII.—OPERA MINORA:
Introductory Address by Dr. Delafield, New York. 80
Relation between the Respiratory and Circulatory Functions, by Dr. Hooker,. 81
Address before the Medical Society of Tennessee, by Professor Yandall. 84
Designs for Marine Hospitals in the West, by Robert Mills. 85
Prize Dissertation on Cholera Infantum, by Dr. King. 85
Statistics of the Blind by Mr. Hazard. 88
Observations for the Red Sulphur Spring, by Dr. Huntt. 91
Memorial on the Advancement of Meteorology, on the plan of Professor Espy. 93
Epidemic Yellow Fever of Natchez, by Dr. Monette. 95

### MISCELLANEOUS INTELLIGENCE.

### ANALECTIC.

ART. I.—Observations on the Puriform Opthalmia of Infants, by Carron du Villards. 107
II.—Inversion of the Uterus, by Mr. Bradford. 111
III.—Nature and Treatment of Nervous Exhaustion, by Dr. Rees 113

*Page.*
IV.—Clinical Lecture, by Mr. Lawrence. 116
V.—Inhalation in Tubercular Phthisis Pulmonalis, by Dr. Scudamore. 121
VI—Medico-Legal considerations on Monomania, by M. Marc, 131

ANALYTICAL.

Art. I—Hydrosudopathia, by M. Fleury. 132
II.—Fractures treated with starched bandages, by Velpeau. 141
III.—Chronic Hydrocephalus cured by a fracture of the skull, by Dr. Hofling. 141
IV.—Quarantine on the Danube, by Miss Pardoe. 142
V.—Present State of Organic Chemistry, by M. Dumas. 147
VI.—Obstetrical Statistics, by Dr. Busch. 149
VII.—London Journals—Creosote in tooth ache, by Mr. Saunders 150

ORIGINAL.

Art. I.—Cincinnati Medical School. 153
II.—The East and West. 156
III.—The Graham Journal of Health and Longevity. 158
IV.—Western Medical Appointments. 162
V.—Medical Journals of the West. 163
VI.—The Black Art. 164
VII.—New Medical School of Virginia. 166
VIII.—Dean's advertisement of Lectures in the Cincinnati College. 167

